# Anti-Inflammatory Activity of the Combination of Nobiletin and Docosahexaenoic Acid in Lipopolysaccharide-Stimulated RAW 264.7 Cells: A Potential Synergistic Anti-Inflammatory Effect

**DOI:** 10.3390/nu16132080

**Published:** 2024-06-29

**Authors:** Kosuke Nishi, Yuki Nakatani, Momoko Ishida, Ayumu Kadota, Takuya Sugahara

**Affiliations:** 1 Food and Health Function Research Center, Ehime University, Matsuyama 790-8566, Japan; nishi.kosuke.mx@ehime-u.ac.jp (K.N.); ishida.momoko.vb@ehime-u.ac.jp (M.I.); 2 Department of Bioscience, Graduate School of Agriculture, Ehime University, Matsuyama 790-8566, Japan; d612031a@mails.cc.ehime-u.ac.jp; 3 Ikata Service Co., Ltd., Ikata 769-0421, Japan; a_noi@ikata-s.co.jp

**Keywords:** nobiletin, docosahexaenoic acid, inflammation, lipopolysaccharide, RAW 264.7 cells, synergism

## Abstract

This study aimed to investigate a synergistic anti-inflammatory effect of a citrus flavonoid nobiletin and docosahexaenoic acid (DHA), one of *n*-3 long-chain polyunsaturated fatty acids, in combination. Simultaneous treatment with nobiletin and DHA synergistically inhibited nitric oxide production (combination index < 0.9) by mouse macrophage-like RAW 264.7 cells stimulated with lipopolysaccharide (LPS) without cytotoxicity. On the other hand, the inhibitory effect of nobiletin and DHA in combination on proinflammatory cytokine production was not synergistic. Neither nobiletin nor DHA affected the phagocytotic activity of RAW 264.7 cells stimulated with LPS. Immunoblot analysis revealed that the inhibition potency of DHA on the phosphorylation of ERK and p38 and nuclear translocation of NF-κB is markedly enhanced by simultaneously treating with nobiletin, which may lead to the synergistic anti-inflammatory effect. Overall, our findings show the potential of the synergistic anti-inflammatory effect of nobiletin and DHA in combination.

## 1. Introduction

Inflammation is one of the defensive immune reactions, which occurs upon injury in the body to eliminate pathogens and damaged cells and to regenerate injured tissues. Macrophages are effector immune cells involved in the promotion and resolution of inflammation [1]. Normally, although the immune system is arranged to be protective, excessive activation of macrophages may lead to severe inflammatory diseases by enormously producing proinflammatory cytokines and chemokines [2]. Generally, inflammation is treated by medications. In recent years, alleviating or preventing inflammation with a daily diet has attracted much attention. Several studies have shown that various food ingredients possess an anti-inflammatory effect [3,4,5,6].

**Figure 1:**
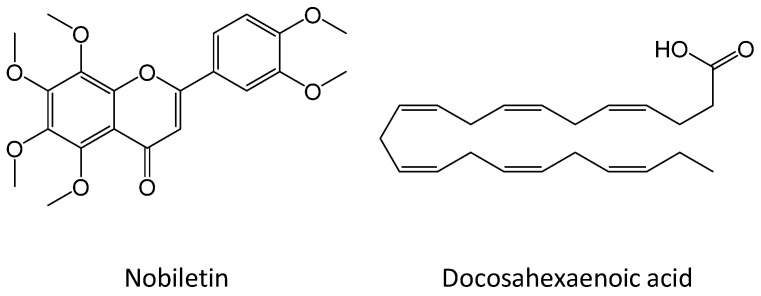
Chemical structures of nobiletin and docosahexaenoic acid.

Synergy is observed when the combined effect of substances is greater than would have been expected from individual contributions, and synergistic effects are vitally important in phytomedicines [7]. Exploiting the synergic effect of food components is also attractive. Synergistic anti-inflammatory effects of plant-derived food components have recently been investigated in detail [8]. We have explored the synergistic anti-inflammatory effect of combined food ingredients because the synergistic action of different food ingredients in combination can be expected to increase the efficacy of anti-inflammatory activities with low doses and reduce side effects. We then discovered a synergistic anti-inflammatory effect of nobiletin (Figure 1) and docosahexaenoic acid or DHA (Figure 1) in combination. Nobiletin, a polymethoxyflavone particularly abundant in citrus peel, has been reported to have an anti-inflammatory effect [9,10]. The synergistic anti-inflammatory effect of nobiletin combined with sulforaphane, an aliphatic isothiocyanate found in vegetables of the family Brassicaceae, has been reported [11]. DHA, an *n*-3 polyunsaturated fatty acid found in oily fish, has also been reported to possess an anti-inflammatory effect [12,13]. DHA has been reported to exert a synergistic anti-inflammatory effect in combination with curcumin, an ingredient of turmeric [14], and with celecoxib, a nonsteroidal anti-inflammatory drug [15]. However, the synergistic anti-inflammatory effect of nobiletin combined with DHA remains unclear. In this paper, we clarified the synergistic anti-inflammatory effect of nobiletin and DHA in combination and elucidated the possible mechanism of the synergistic action.

## 2. Materials and Methods

### 2.1. Reagents

Nobiletin and DHA were obtained from Fujifilm Wako Pure Chemical (Osaka, Japan) and Cayman Chemical (Ann Arbor, MI, USA), respectively. Nobiletin was dissolved in dimethyl sulfoxide, while DHA was dissolved in ethanol. Fetal bovine serum (FBS), lipopolysaccharide (LPS) from *Escherichia coli* 026/B6, streptomycin, penicillin, and Dulbecco’s modified Eagle’s medium (DMEM) were purchased from Sigma-Aldrich (St. Louis, MO, USA). Horseradish peroxidase (HRP)-labeled anti-rabbit IgG antibody and rabbit monoclonal antibodies against p38 mitogen-activated protein (MAP) kinase, phosphorylated p38 MAP kinase (p-p38), c-Jun *N*-terminal kinases (JNK), phosphorylated JNK (p-JNK), extracellular signal-regulated kinases (ERK) 1/2, phosphorylated ERK1/2 (p-ERK1/2), lamin B1, glyceraldehyde-3-phosphate dehydrogenase (GAPDH), and nuclear factor kappa B (NF-κB) p65 were obtained from Cell Signaling Technology (Danvers, MA, USA). All other chemicals were obtained from Nacalai Tesque (Kyoto, Japan) or Fujifilm Wako Pure Chemical, unless otherwise noted.

### 2.2. Cell Culture

Mouse macrophage-like cell line RAW 264.7 cells were obtained from the European Collection of Authenticated Cell Cultures (London, UK) and cultured in DMEM supplemented with 10% FBS, 100 µg/mL of streptomycin, and 100 U/mL of penicillin at 37 °C under humidified 5% CO_2_ in air.

### 2.3. Griess Assay

RAW 264.7 cells were seeded at 2.0 × 10^4^ cells/well in a 96-well culture plate and precultured for 18 h. After removing the culture medium, fresh DMEM containing samples and LPS (final concentration: 10 µg/mL) was added to each well of the plate, and the cells were cultured for 24 h. The culture medium was then used for measuring the concentration of nitrite, an oxidized form of nitric oxide, using the Griess Reagent System (Madison, WI, Promega) according to the manufacturer’s protocol.

### 2.4. Combination Index (CI) Calculation

The effect of nobiletin and DHA in combination on nitric oxide production by RAW 264.7 cells stimulated with LPS was analyzed by the CI value to define whether the combination provided a synergistic, additive, or antagonistic effect. The CI value was calculated according to previous studies [16,17] based on the Chou–Talalay method [18,19]. The calculation was conducted using the following formula:

(1) $$CI=\frac{{IC}_{50_{Nobiletin–comb}}}{{IC}_{50_{Nobiletin}}}+\frac{{IC}_{50_{DHA–comb}}}{{IC}_{50_{DHA}}}$$

A CI of <0.9 was considered synergistic, a CI of ≥0.9 and ≤1.1 was considered additive, and a CI of >1.1 was considered antagonistic [20,21,22]. Median inhibitory concentration (IC_50_) values of individual and combined compounds were determined based on the dose–response curve.

### 2.5. WST-8 Assay

Cytotoxicity of samples to RAW 264.7 cells was evaluated by the WST-8 assay using Cell Count Reagent (Nacalai Tesque). RAW 264.7 cells were seeded at 2.0 × 10^4^ cells/well into a 96-well culture plate and precultured for 18 h. After removing the culture medium, fresh DMEM containing samples and LPS (final concentration: 1 µg/mL) was added to each well of the plate, and the cells were cultured for 6 h. After removing the culture medium, fresh DMEM containing 10% Cell Count Reagent was added. The absorbance was then measured at 450 nm using a microplate reader (iMark, Bio-Rad Laboratories, Hercules, CA, USA).

### 2.6. Phagocytosis Assay

A phagocytosis assay was performed as previously reported [23]. RAW 264.7 cells were seeded at 6.0 × 10^5^ cells/well in a 6-well culture plate and precultured for 18 h. After removing the culture medium, fresh DMEM containing 13 µM nobiletin and/or 20 µM DHA was added to each well of the plate, and the cells were stimulated with 1 µg/mL of and LPS for 15 min. After washing the cells with phosphate-buffered saline (PBS, pH 7.4), fresh DMEM containing 20 mg/mL of Zymosan A (Saccharomyces cerevisiae) labeled with Texas Red was added, and the cells were incubated for 1 h in the dark. After removing the culture medium, the cells were washed with PBS and collected. After centrifugation at 350× *g* for 5 min, the collected cells were suspended in 1 mL of PBS containing 2% FBS, and the percentage of Texas Red-positive cells was measured using a flow cytometer (FACSCalibur, BD Biosciences, San Jose, CA, USA).

### 2.7. Cytokine Measurement

RAW 264.7 cells were seeded at 2.0 × 10^4^ cells/well in a 96-well culture plate and precultured for 18 h. After removing the culture medium, fresh DMEM containing 13 µM nobiletin and/or 20 µM DHA and LPS (final concentration: 1 µg/mL) was added to each well of the plate, and the cells were cultured for 24 h. The culture medium was collected, and proinflammatory cytokine concentrations were determined by enzyme-linked immunosorbent assay (ELISA) kits for mouse interleukin (IL)-1β and IL-6 (BioLegend, San Diego, CA, USA) and for mouse tumor necrosis factor α (TNF-α) (Thermo Fisher Scientific, Waltham, MA, USA) according to the manufacturer’s protocol.

### 2.8. Real-Time RT-PCR

RAW 264.7 cells were seeded at 2.0 × 10^5^ cells/well in a 24-well culture plate and precultured for 18 h. After removing the culture medium, fresh DMEM containing 13 µM nobiletin and/or 20 µM DHA was added to each well of the plate, and the cells were stimulated with 1 µg/mL of LPS for 3 h. Total RNA was extracted from the cells using Sepasol-RNA I Super G (Nacalai Tesque). The RNA was used for cDNA synthesis using M-MLV reverse transcriptase (Nippon Gene, Tokyo, Japan) and an oligo dT_20_ primer (Toyobo, Osaka, Japan). Real-time PCR was conducted using Thunderbird SYBR qPCR Mix (Toyobo), a forward primer, and a reverse primer by repeating 40 cycles of thermal cycling conditions of 95 °C for 3 s and 60 °C for 30 s after 95 °C for 20 s. PCR products were measured using the StepOnePlus Real-Time PCR System (Applied Biosystems, Foster City, CA, USA), and analysis was performed with StepOne Software v2.1 (Applied Biosystems). Relative gene expression was determined by the comparative Ct method using β-actin as a reference gene. The nucleotide sequences of specific primers used are as follows: mouse nitric oxide synthase 2 (NOS2); sense, 5′-CCAAGGCCTCACCTACTTCC-3′, and antisense, 5′-CTCTGAGGGCTGACACAAGG-3′; mouse β-actin: sense, 5′-CATCCGTAAAGACCTCTATGCCAAC-3′, and antisense, 5′-ATGGAGCCACCGATCCACA-3′.

### 2.9. Immunostaining

RAW 264.7 cells were immunostained to assess NOS2 expression by flow cytometry according to the manufacturer’s protocol. RAW 264.7 cells were seeded at 6.0 × 10^5^ cells/well in a 6-well culture plate and precultured for 18 h. After removing the culture medium, fresh DMEM containing 13 µM nobiletin and/or 20 µM DHA and LPS (final concentration: 1 µg/mL) was added to each well of the plate, and the cells were cultured for 24 h. After removing the culture medium, the cells were washed with PBS and collected. After centrifugation at 350× *g* for 5 min, the cells were permeabilized with pre-chilled methanol for 15 min. After washing the cells with PBS containing 2% FBS, immunostaining was performed with an anti-NOS2 antibody (1:1000 dilution) labeled with phycoerythrin (PE) (Cell Signaling Technology) at room temperature for 20 min under a dark condition. After washing, the cells were suspended in 0.5 mL of PBS containing 2% FBS, and the percentage of PE-positive cells was measured using a FACSCalibur flow cytometer (BD Biosciences) to assess NOS2 expression in RAW 264.7 cells.

### 2.10. Immunoblot Analysis

RAW 264.7 cells were seeded at 6.0 × 10^5^ cells/well in a 6-well culture plate and precultured for 18 h. After removing the culture medium, fresh DMEM containing 13 µM nobiletin and/or 20 µM DHA was added to each well of the plate, and the cells were stimulated with 1 µg/mL of LPS for 15 min. Nuclear and cytosolic proteins were prepared using the CelLytic NuCLEAR Extraction Kit (Sigma-Aldrich) according to the manufacturer’s protocol. Proteins were separated by sodium dodecyl sulfate-polyacrylamide gel electrophoresis and transferred to polyvinylidene difluoride membranes (Hybond-P; GE Healthcare, Buckinghamshire, UK). The membranes were then blocked with Blocking One (Nacalai Tesque) or Blocking One-P (Nacalai Tesque) at room temperature for 1 h and subsequently reacted with each primary antibody overnight at 4 °C. Antibodies were diluted according to the manufacturer’s recommended dilution ratio. After washing, the membranes were reacted with a secondary antibody labeled with HRP at room temperature for 1 h. After washing, the membranes were developed with ImmnoStar LD (Fujifilm Wako Pure Chemical), and chemiluminescence was detected using a ChemiDoc XRS Plus apparatus (Bio-Rad Laboratories) with Image Lab software version 2.0.1 (Bio-Rad Laboratories).

### 2.11. Statistical Analysis

Statistical analyses were performed using GraphPad Prism version 7.05 (GraphPad Software, Boston, MA, USA). Statistical significance was determined via one-way analysis of variance with Dunnett’s test or Tukey’s test as indicated. The significance level used was *p* < 0.05.

## 3. Results and Discussion

### 3.1. Synergistic Inhibitory Effect of Nobiletin and DHA in Combination on Nitric Oxide Production by LPS-Stimulated RAW 264.7 Cells

**Figure 2:**
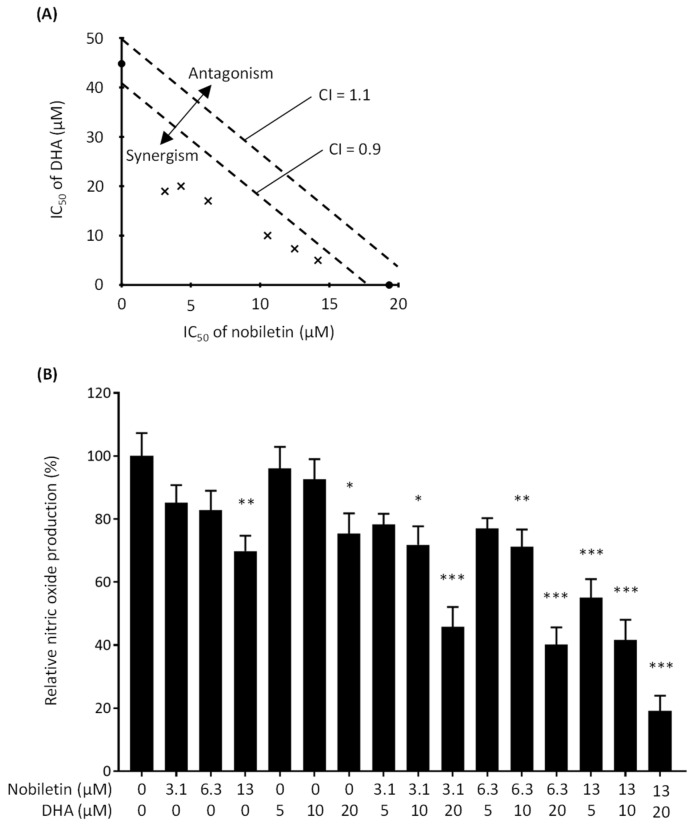
Effect of nobiletin and docosahexaenoic acid (DHA) on nitric oxide production by lipopolysaccharide (LPS)-stimulated RAW 264.7 cells. (**A**) Isobologram showing the type of interaction produced by nobiletin and DHA. Closed circles indicate the IC_50_ values of nobiletin or DHA alone. Crosses indicate the IC_50_ values of nobiletin or DHA in combination. (**B**) Relative nitric oxide production by LPS-stimulated RAW 264.7 cells treated with nobiletin alone, DHA alone, and the combination of nobiletin and DHA. Data are expressed as the mean ± SEM (*n* = 6). * *p* < 0.05, ** *p* < 0.01, and *** *p* < 0.001 against the control (no treatment with nobiletin or DHA) by Dunnett’s test.

We screened combinations of various food ingredients using RAW 264.7 cells for a synergistic anti-inflammatory effect. The cells were stimulated with LPS, a potent inducer, to secret proinflammatory mediators, such as IL-6, and reactive oxygen species, such as nitric oxide, from macrophages. At first, we evaluated the effect of combinations of various food-derived molecules on nitric oxide production by a Griess assay. Griess reagent reacts with nitrite, which was used as a measure of nitric oxide production during the assay. As a result of screening, we found a synergistic inhibitory effect of the simultaneous treatment with nobiletin and DHA on nitric oxide production. Nitric oxide is a signaling molecule made from L-arginine by NOS present in various tissues. Nitric oxide produced by macrophages plays a crucial role in the onset of inflammation; nitric oxide produced in excess is a proinflammatory mediator. First, we determined the IC_50_ values of each compound alone for nitric oxide production by a Griess assay. The result showed that the IC_50_ value of nobiletin alone was 19 µM, while that of DHA alone was 45 µM (Figure 2A). This tendency was observed in previous studies showing the inhibitory potency of nobiletin [24,25] and DHA [26,27] on nitric oxide production.

Next, we performed a Griess assay with various concentrations of DHA and nobiletin in combination and determined IC_50_ values to assess whether the simultaneous action of both compounds is a synergism (Figure 2B). When nobiletin concentration was fixed at 13 µM, the IC_50_ value was obtained with 7.3 µM DHA (CI = 0.81), as shown in Figure 2A. In the same manner, the IC_50_ values were obtained with 19 and 17 µM DHA with the CI values of 0.58 and 0.70 when nobiletin concentrations were fixed at 3.1 and 6.3 µM, respectively (Figure 2A). On the other hand, the IC_50_ values were obtained with 14, 11, and 4.3 µM nobiletin with the CI values of 0.85, 0.77, and 0.69 when DHA concentrations were fixed at 5, 10, and 20 µM, respectively (Figure 2A). The result revealed that simultaneous treatment of both nobiletin and DHA markedly inhibits NO production. Because all calculated CI values were lower than 0.9, the simultaneous action of nobiletin and DHA on NO production was defined as synergism.

**Figure 3:**
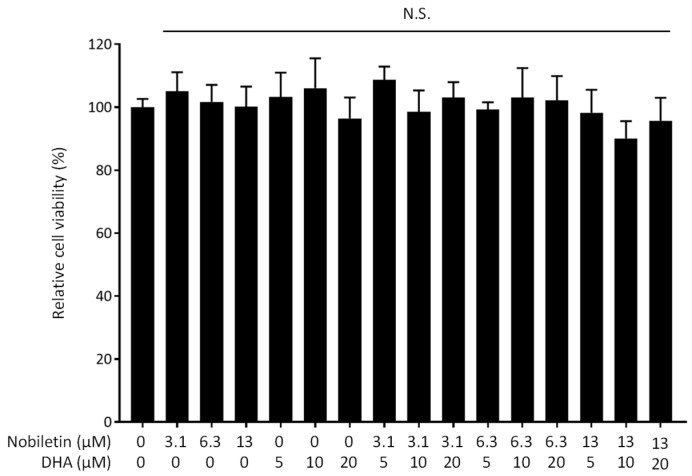
Effect of nobiletin and docosahexaenoic acid (DHA) on the viability of RAW 264.7 cells. Data are expressed as the mean ± SEM (*n* = 6). N.S. indicates no statistical significance against the control (no treatment with nobiletin or DHA) by Dunnett’s test.

The cytotoxicity of nobiletin and DHA to RAW 264.7 cells was assessed by a WST-8 assay. No reduction in cell viability was observed in any concentration range investigated (Figure 3). We thus concluded that nobiletin and DHA in combination exhibit a synergistic inhibitory effect on NO production without cytotoxicity.

### 3.2. Effect of Nobiletin and DHA in Combination on the Phagocytosis of LPS-Stimulated RAW 264.7 Cells

**Figure 4:**
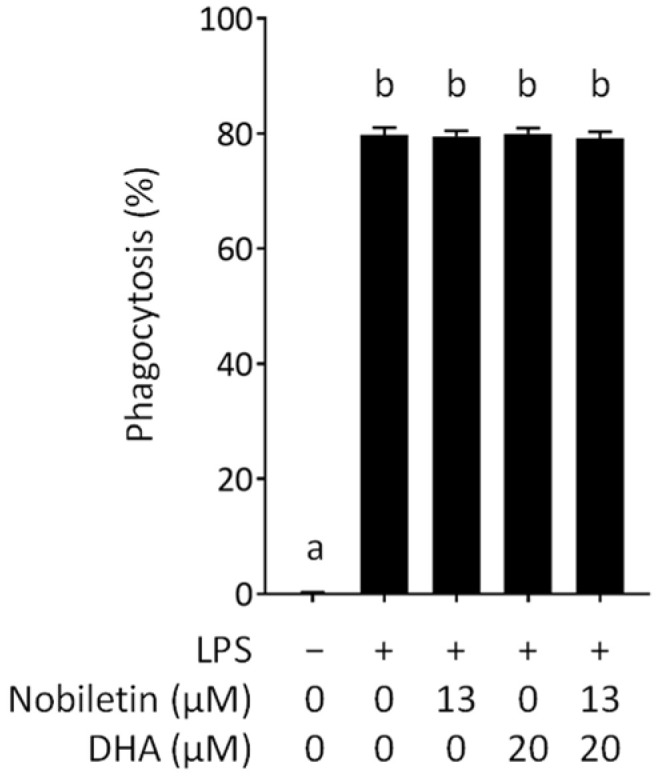
Effect of nobiletin and docosahexaenoic acid (DHA) on the phagocytotic capacity of lipopolysaccharide-stimulated RAW 264.7 cells. Phagocytosis (%) means the percentage of phagocytosed cells. Different letters above the bars indicate significant differences by Tukey’s test (*p* < 0.05).

We assessed the effect of nobiletin and DHA on the phagocytotic activity of RAW 264.7 cells stimulated with LPS because phagocytosis is one of the key reactions of macrophages for the onset of the innate immune response [28]. After phagocytosis, macrophages present antigens and secret proinflammatory proteins in response to invading pathogens in the body. Antigen presentation of phagocytosed pathogens as an antigen on the cell surface activates the adaptive immune system. We evaluated the phagocytosis rate by measuring the percentage of Texas Red-positive RAW 264.7 cells on a flow cytometer. As a result, we found that nobiletin or DHA alone does not affect the phagocytotic capacity of RAW 264.7 cells (Figure 4). The effect of DHA on the phagocytotic activity of macrophages is still controversial. A research group reported the reduced phagocytotic activity of rat peritoneal macrophages by DHA treatment [29], whereas another group reported the enhanced phagocytotic capacity of RAW 264.7 cells by DHA treatment [30]. Our result was consistent with the data of Lokesh and Kinsella (1987), who reported that DHA does not affect the phagocytotic activity of mouse peritoneal macrophages [31]. On the other hand, the effect of nobiletin on the phagocytic capacity of macrophages remains unknown. For the first time, we revealed that nobiletin does not affect the phagocytotic activity of RAW 264.7 cells (Figure 4) despite its anti-inflammatory activity. We also assessed the effect of nobiletin and DHA in combination on the phagocytosis of RAW 264.7 cells. The data showed that the combination of nobiletin and DHA does not change the phagocytotic activity (Figure 4).

### 3.3. Inhibitory Effect of Nobiletin and DHA in Combination on the Secretion of Proinflammatory Cytokines from LPS-Stimulated RAW 264.7 Cells

**Figure 5:**
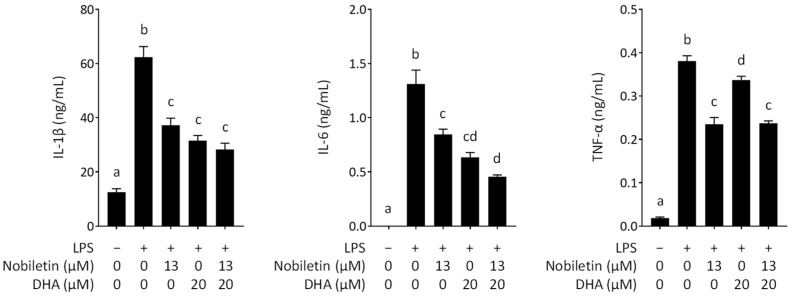
Effect of nobiletin and docosahexaenoic acid (DHA) on the proinflammatory cytokine secretion by lipopolysaccharide (LPS)-stimulated RAW 264.7 cells. Data are expressed as the mean ± SEM (*n* = 6). Different letters above the bars indicate significant differences by Tukey’s test (*p* < 0.05).

LPS, which consists of lipids and carbohydrates present on the cell wall surface of Gram-negative bacteria, is a macrophage activator and causes macrophages to secrete large amounts of various proinflammatory cytokines, including IL-1β, IL-6, and TNF-α [32,33,34]. We assessed the effect of nobiletin and DHA on the secretion of proinflammatory cytokines from RAW 264.7 cells stimulated with LPS by ELISAs. The anti-inflammatory activities of nobiletin and DHA alone were confirmed, as shown in Figure 5. The data were consistent with those in previous papers, which showed the inhibitory activities of nobiletin and DHA on the production of proinflammatory cytokines [10,13]. We then assessed the effect of nobiletin and DHA in combination. The result showed that the combination of nobiletin and DHA enhanced the inhibitory effect of proinflammatory cytokine secretion; however, the effect was not synergistic (Figure 5). We thus concluded that the combination of DHA and nobiletin exerts a synergistic anti-inflammatory effect on NO production but not on the proinflammatory cytokine secretion from RAW 264.7 cells stimulated with LPS.

### 3.4. Effect of Nobiletin and DHA in Combination on NOS2 Expression in LPS-Stimulated RAW 264.7 Cells

**Figure 6:**
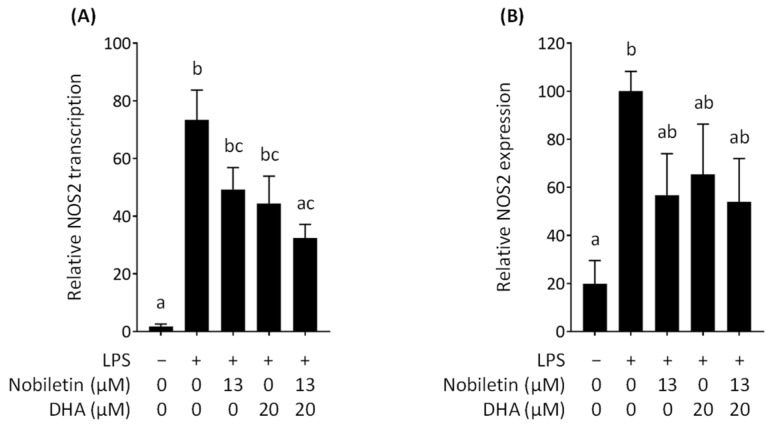
Effect of nobiletin and docosahexaenoic acid (DHA) on NOS2 expression in lipopolysaccharide (LPS)-stimulated RAW 264.7 cells. Data are expressed as the mean ± SEM (*n* = 6). Different letters above the bars indicate significant differences by Tukey’s test (*p* < 0.05). (**A**) Relative NOS2 transcription measured by real-time PCR. (**B**) Relative NOS2 translation measured by flow cytometry.

Nitric oxide is synthesized by the enzyme NOS2, also commonly called iNOS, in macrophages [35]. NOS2 expression is induced by an inflammatory stimulus such as LPS. NOS2, once expressed in macrophages, can abundantly produce nitric oxide for the host defense to kill the invading pathogens. Because the combination of nobiletin and DHA synergistically inhibited nitric oxide production by RAW 264.7 cells stimulated with LPS (Figure 2A), we evaluated the effect of nobiletin and DHA on the NOS2 transcription by real-time RT-PCR. The result showed that nobiletin downregulates the transcription of *Nos2* (Figure 6A), which was also observed in previous studies [25,36,37]. We also found that DHA reduces the transcription of *Nos2* (Figure 6A), which was also consistent with the data shown in previous reports [38,39]. The combination of nobiletin and DHA inhibited *Nos2* transcription stronger than each compound alone (Figure 6A); however, the inhibitory effect was not synergistic.

The protein level of NOS2 was assessed with LPS-stimulated RAW 264.7 cells stained with an anti-NOS2 antibody labeled with PE by flow cytometry. The result showed that treating the cells with nobiletin decreased the NOS2 protein level (Figure 6B), which was found in the previous papers [40,41,42]. DHA also decreased the NOS2 protein amount (Figure 6B), which is consistent with the data obtained in a previous study [26]. Nobiletin and DHA in combination inhibited the NOS2 protein level stronger than each compound alone (Figure 6B); however, the inhibitory effect of the combination was again not synergistic. These results suggest that the simultaneous action of nobiletin and DHA synergistically suppresses nitric oxide production in part, but not entirely, because of the downregulated NOS2 expression in RAW 264.7 cells stimulated with LPS. Zhang et al. (2022a) also reported that the NOS2 protein level was not decreased synergistically by the combination of camellia oil and proanthocyanidin from lipophilic grape seeds, although nitric oxide production was synergistically inhibited by the combination in RAW 264.7 cells stimulated with LPS [43]. Other previous studies showed synergistic inhibition for nitric oxide production in macrophages by the combination of food ingredients; however, these papers showed no data on NOS2 expression [44,45]. Therefore, it would be difficult to observe a distinct difference in the transcriptional or translational level of NOS2 even though a synergistic inhibitory effect on nitric oxide production was found. The inhibited catalytic reaction of NOS2 by either nobiletin or DHA can be considered a potential mechanism for the synergistic effect. Further investigation on the potential inhibitory activity of nobiletin and DHA on the enzymatic activity of NOS2 is needed.

### 3.5. Effect of Nobiletin and DHA in Combination on Intracellular Signal Transduction in LPS-Stimulated RAW 264.7 Cells

**Figure 7:**
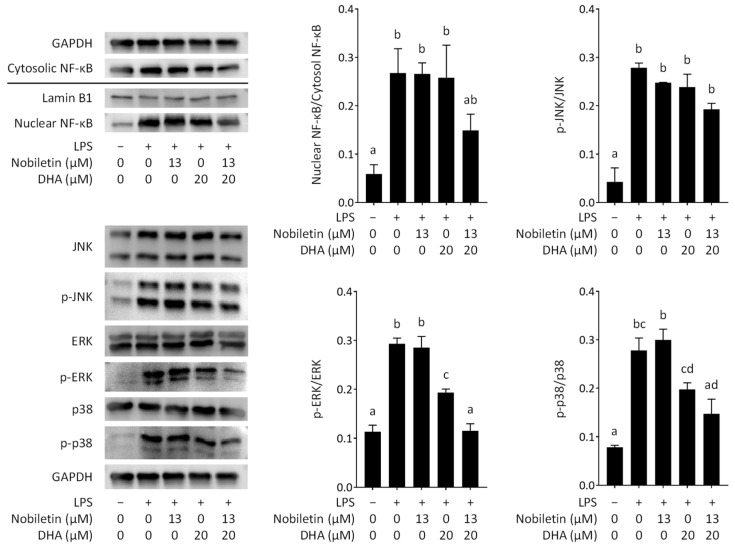
Effect of docosahexaenoic acid (DHA) and nobiletin on intracellular signaling in lipopolysaccharide (LPS)-stimulated RAW 264.7 cells assessed by immunoblot analysis. p-JNK, p-ERK, and p-p38 represent phosphorylated JNK, phosphorylated ERK, and phosphorylated p38 MAP kinase, respectively. A representative blot from three independent experiments is shown. Data are expressed as the mean ± SEM (*n* = 3). Different letters above the bars indicate significant differences by Tukey’s test (*p* < 0.05).

Macrophages receive various stimuli, such as LPS, from outside the cell with receptors on the plasma membrane and transmit the information inside the cell to perform an appropriate response. LPS activates NF-κB and MAP kinase signaling via Toll-like receptor 4 (TLR4). TLR4 activation induces IKK phosphorylation, and the phosphorylated IKK in turn phosphorylates IκBα. NF-κB then disassociates from IκBα and translocates into the nucleus to transcribe proinflammatory cytokine genes as a transcription factor. Transcriptional induction of NOS2 is largely dependent on the activation of MAP kinase and NF-κB pathways [46,47]. We thus assessed the effect of nobiletin and DHA on the nuclear translocation of NF-κB. The amounts of NF-κB p65 in the nucleus and cytosol were each detected by immunoblotting. The result showed that nobiletin or DHA alone has little effect on the NF-κB nuclear translocation; however, nobiletin and DHA in combination inhibited the NF-κB nuclear translocation much more than the addition of inhibitory effects by each of both compounds, although the suppressive effect of the combination was not statistically significant against the control (LPS stimulation only), as shown in Figure 7. It was thus suggested that the simultaneous action of nobiletin and DHA might synergistically suppress NO production by enhancing their inhibitory potency for the nuclear translocation of NF-κB. Zhang et al. (2022b) reported that curcumin and resveratrol in combination exert a synergistic anti-inflammatory activity and that nuclear translocation of NF-κB p65 is inhibited by curcumin alone but not by resveratrol alone [48]. They also showed in their study that the inhibitory potency of curcumin for the nuclear translocation of NF-κB p65 was significantly strengthened by the combined treatment with resveratrol. The effect of nobiletin and DHA in combination on NF-κB nuclear translocation was thus quite similar to that of resveratrol and curcumin in combination, which indicates a potential common mechanism underlying the synergistic anti-inflammatory effects.

The MAP kinase family consists of ERK, JNK, and p38 MAP kinase. MAP kinase signaling is activated by a cascade of protein phosphorylation reactions and is involved in the expression of proinflammatory genes. We also evaluated the effect of the simultaneous action of nobiletin and DHA on the phosphorylation of MAP kinases. The result showed that nobiletin and DHA in combination inhibited the phosphorylation of JNK as much as the addition of suppressive effects by each compound (Figure 7). On the other hand, the phosphorylation of ERK and p38 MAP kinase was inhibited with 20 µM DHA but not with 13 µM nobiletin. Interestingly, the phosphorylation of p38 MAP kinase and ERK was significantly enhanced by combining DHA with nobiletin (Figure 7). Chen et al. also reported that silibinin and thymol in combination exert an anti-inflammatory effect and that the phosphorylation of p38 MAP kinase and ERK was synergistically inhibited, whereas that of JNK was additively suppressed [49]. Park et al. showed an anti-inflammatory effect of aconitine and methotrexate in combination via synergistically inhibited phosphorylation of ERK [50]. Thus, our data suggested that nobiletin and DHA might synergistically suppress nitric oxide production through enhanced downregulation of phosphorylation of ERK and p38.

## 4. Conclusions

We found a synergistic anti-inflammatory effect of nobiletin and DHA on nitric oxide production by RAW 264.7 cells stimulated with LPS. Neither nobiletin nor DHA affected the phagocytotic capacity of RAW 264.7 cells stimulated with LPS. Immunoblot analysis revealed that nobiletin and DHA synergistically inhibit NF-κB nuclear translocation and the phosphorylation of p38 MAP kinase and ERK, which may lead to the synergistic anti-inflammatory effect. Overall, our findings show the potential of the synergistic anti-inflammatory effect of DHA and nobiletin in combination, which can be applied to the development of novel functional foods for the alleviation of inflammatory reactions.

## Data Availability

Data are contained within the article.

## References

[1] Koh T.J. (2011). Inflammation and wound healing: The role of the macrophage. Expert Rev. Mol. Med..

[2] Garlanda C., Di Liberto D., Vecchi A., La Manna M.P., Buracchi C., Caccamo N., Salerno A., Dieli F., Mantovani A. (2007). Damping excessive inflammation and tissue damage in *Mycobacterium tuberculosis* infection by Toll IL-1 receptor 8/single Ig IL-1-related receptor, a negative regulator of IL-1/TLR signaling. J. Immunol..

[3] Dell’Agli M., Di Lorenzo C., Badea M., Sangiovanni E., Dima L., Bosisio E., Restani P. (2013). Plant food supplements with anti-inflammatory properties: A systematic review (I). Crit. Rev. Food Sci. Nutr..

[4] Lu C.C., Yen G.C. (2015). Antioxidative and anti-inflammatory activity of functional foods. Curr. Opin. Food Sci..

[5] Serafini M., Peluso I. (2016). Functional foods for health: The interrelated antioxidant and anti-inflammatory role of fruits, vegetables, herbs, spices and cocoa in humans. Curr. Pharm. Des..

[6] Di Lorenzo C., Dell’Agli M., Badea M., Dima L., Colombo E., Sangiovanni E., Restani P., Bosisio E. (2013). Plant food supplements with anti-inflammatory properties: A systematic review (II). Crit. Rev. Food Sci. Nutr..

[7] Williamson E.M. (2001). Synergy and other interactions in phytomedicines. Phytomedicine.

[8] Zhang L., Virgous C., Si H. (2019). Synergistic anti-inflammatory effects and mechanisms of combined phytochemicals. J. Nutr. Biochem..

[9] Murakami A., Nakamura Y., Ohto Y., Yano M., Koshiba T., Koshimizu K., Tokuda H., Nishino H., Ohigashi H. (2000). Suppressive effects of citrus fruits on free radical generation and nobiletin, an anti-inflammatory polymethoxyflavonoid. Biofactors.

[10] Lin N., Sato T., Takayama Y., Mimaki Y., Sashida Y., Yano M., Ito A. (2003). Novel anti-inflammatory actions of nobiletin, a citrus polymethoxy flavonoid, on human synovial fibroblasts and mouse macrophages. Biochem. Pharmacol..

[11] Guo S., Qiu P., Xu G., Wu X., Dong P., Yang G., Zheng J., McClements D.J., Xiao H. (2012). Synergistic anti-inflammatory effects of nobiletin and sulforaphane in lipopolysaccharide-stimulated RAW 264.7 cells. J. Agric. Food Chem..

[12] Raederstorff D., Pantze M., Bachmann H., Moser U. (1996). Anti-inflammatory properties of docosahexaenoic and eicosapentaenoic acids in phorbol-ester-induced mouse ear inflammation. Int. Arch. Allergy Immunol..

[13] Weldon S.M., Mullen A.C., Loscher C.E., Hurley L.A., Roche H.M. (2007). Docosahexaenoic acid induces an anti-inflammatory profile in lipopolysaccharide-stimulated human THP-1 macrophages more effectively than eicosapentaenoic acid. J. Nutr. Biochem..

[14] Saw C.L., Huang Y., Kong A.N. (2010). Synergistic anti-inflammatory effects of low doses of curcumin in combination with polyunsaturated fatty acids: Docosahexaenoic acid or eicosapentaenoic acid. Biochem. Pharmacol..

[15] Kondreddy V.K., Kamatham A.N. (2016). Celecoxib, a COX-2 inhibitor, synergistically potentiates the anti-inflammatory activity of docosahexaenoic acid in macrophage cell line. Immunopharmacol. Immunotoxicol..

[16] Wisniewski-Rebecca E.S., Rocha B.A., Wiirzler L.A., Cuman R.K., Velazquez-Martinez C.A., Bersani-Amado C.A. (2015). Synergistic effects of anethole and ibuprofen in acute inflammatory response. Chem. Biol. Interact..

[17] Vissiennon C., Hammoud D., Goos K.H., Nieber K., Arnhold J. (2017). Synergistic interactions of chamomile flower, myrrh and coffee charcoal in inhibiting pro-inflammatory chemokine release from activated human macrophages. Synergy.

[18] Chou T.C., Talalay P. (1984). Quantitative analysis of dose-effect relationships: The combined effects of multiple drugs or enzyme inhibitors. Adv. Enzyme Regul..

[19] Chou T.C. (2010). Drug combination studies and their synergy quantification using the Chou-Talalay method. Cancer Res..

[20] Brown P., Levis M., McIntyre E., Griesemer M., Small D. (2006). Combinations of the FLT3 inhibitor CEP-701 and chemotherapy synergistically kill infant and childhood MLL-rearranged ALL cells in a sequence-dependent manner. Leukemia.

[21] Tardi P., Johnstone S., Harasym N., Xie S., Harasym T., Zisman N., Harvie P., Bermudes D., Mayer L. (2009). *In vivo* maintenance of synergistic cytarabine:daunorubicin ratios greatly enhances therapeutic efficacy. Leuk. Res..

[22] Férir G., Palmer K.E., Schols D. (2011). Synergistic activity profile of griffithsin in combination with tenofovir, maraviroc and enfuvirtide against HIV-1 clade C. Virology.

[23] Tagashira A., Nishi K., Matsumoto S., Sugahara T. (2018). Anti-inflammatory effect of lysozyme from hen egg white on mouse peritoneal macrophages. Cytotechnology.

[24] Li W., Zhao R., Wang X., Liu F., Zhao J., Yao Q., Zhi W., He Z., Niu X. (2018). Nobiletin-ameliorated lipopolysaccharide-induced inflammation in acute lung injury by suppression of NF-κB pathway in vivo and vitro. Inflammation.

[25] Rong X., Xu J., Jiang Y., Li F., Chen Y., Dou Q.P., Li D. (2021). Citrus peel flavonoid nobiletin alleviates lipopolysaccharide-induced inflammation by activating IL-6/STAT3/FOXO3a-mediated autophagy. Food Funct..

[26] Komatsu W., Ishihara K., Murata M., Saito H., Shinohara K. (2003). Docosahexaenoic acid suppresses nitric oxide production and inducible nitric oxide synthase expression in interferon-gamma plus lipopolysaccharide-stimulated murine macrophages by inhibiting the oxidative stress. Free Radic. Biol. Med..

[27] Choi E.Y., Jin J.Y., Choi J.I., Choi I.S., Kim S.J. (2014). DHA suppresses *Prevotella intermedia* lipopolysaccharide-induced production of proinflammatory mediators in murine macrophages. Br. J. Nutr..

[28] Aderem A., Underhill D.M. (1999). Mechanisms of phagocytosis in macrophages. Annu. Rev. Immunol..

[29] Bulbul M., Tan R., Gemici B., Hacioglu G., Agar A., Izgut-Uysal V.N. (2007). Effect of docosahexaenoic acid on macrophage functions of rats. Immunobiology.

[30] Chang H.Y., Lee H.N., Kim W., Surh Y.J. (2015). Docosahexaenoic acid induces M2 macrophage polarization through peroxisome proliferator-activated receptor γ activation. Life Sci..

[31] Lokesh B.R., Kinsella J.E. (1987). Modulation of prostaglandin synthesis in mouse peritoneal macrophages by enrichment of lipids with either eicosapentaenoic or docosahexaenoic acids in vitro. Immunobiology.

[32] Bonta I.L., Ben-Efraim S., Mózes T., Fieren M.W. (1991). Tumour necrosis factor in inflammation: Relation to other mediators and to macrophage antitumour defence. Pharmacol. Res..

[33] Joosten L.A., Netea M.G., Dinarello C.A. (2013). Interleukin-1β in innate inflammation, autophagy and immunity. Semin. Immunol..

[34] Scheller J., Garbers C., Rose-John S. (2014). Interleukin-6: From basic biology to selective blockade of pro-inflammatory activities. Semin. Immunol..

[35] MacMicking J., Xie Q.W., Nathan C. (1997). Nitric oxide and macrophage function. Annu. Rev. Immunol..

[36] Yoshigai E., Machida T., Okuyama T., Mori M., Murase H., Yamanishi R., Okumura T., Ikeya Y., Nishino H., Nishizawa M. (2013). Citrus nobiletin suppresses inducible nitric oxide synthase gene expression in interleukin-1β-treated hepatocytes. Biochem. Biophys. Res. Commun..

[37] Xie L., Xie H., Chen C., Tao Z., Zhang C., Cai L. (2019). Inhibiting the PI3K/AKT/NF-κB signal pathway with nobiletin for attenuating the development of osteoarthritis: In vitro and in vivo studies. Food Funct..

[38] Khair-El-Din T., Sicher S.C., Vazquez M.A., Chung G.W., Stallworth K.A., Kitamura K., Miller R.T., Lu C.Y. (1996). Transcription of the murine iNOS gene is inhibited by docosahexaenoic acid, a major constituent of fetal and neonatal sera as well as fish oils. J. Exp. Med..

[39] Lu C.Y., Penfield J.G., Khair-el-Din T.A., Sicher S.C., Kielar M.L., Vazquez M.A., Che L. (1998). Docosahexaenoic acid, a constituent of fetal and neonatal serum, inhibits nitric oxide production by murine macrophages stimulated by IFNγ plus LPS, or by IFNγ plus *Listeria monocytogenes*. J. Reprod. Immunol..

[40] Choi S.Y., Hwang J.H., Ko H.C., Park J.G., Kim S.J. (2007). Nobiletin from citrus fruit peel inhibits the DNA-binding activity of NF-κB and ROS production in LPS-activated RAW 264.7 cells. J. Ethnopharmacol..

[41] Xiong Y., Chen D., Yu C., Lv B., Peng J., Wang J., Lin Y. (2015). Citrus nobiletin ameliorates experimental colitis by reducing inflammation and restoring impaired intestinal barrier function. Mol. Nutr. Food Res..

[42] Qi G., Mi Y., Fan R., Li R., Liu Z., Liu X. (2019). Nobiletin protects against systemic inflammation-stimulated memory impairment via MAPK and NF-κB signaling pathways. J. Agric. Food Chem..

[43] Zhang L., Chen J., Liang R., Liu C., Chen M., Chen J. (2022). Synergistic anti-inflammatory effects of lipophilic grape seed proanthocyanidin and camellia oil combination in LPS-stimulated RAW264.7 cells. Antioxidants.

[44] Genito C.J., Eckshtain-Levi M., Piedra-Quintero Z.L., Krovi S.A., Kroboth A., Stiepel R.T., Guerau-de-Arellano M., Bachelder E.M., Ainslie K.M. (2021). Dexamethasone and fumaric acid ester conjugate synergistically inhibits inflammation and NF-κB in macrophages. Bioconjug. Chem..

[45] Zhou X., Afzal S., Wohlmuth H., Münch G., Leach D., Low M., Li C.G. (2022). Synergistic anti-inflammatory activity of ginger and turmeric extracts in inhibiting lipopolysaccharide and interferon-γ-induced proinflammatory mediators. Molecules.

[46] Kim A.R., Lee M.S., Shin T.S., Hua H., Jang B.C., Choi J.S., Byun D.S., Utsuki T., Ingram D., Kim H.R. (2011). Phlorofucofuroeckol A inhibits the LPS-stimulated iNOS and COX-2 expressions in macrophages via inhibition of NF-κB, Akt, and p38 MAPK. Toxicol. In Vitro.

[47] Kim E.A., Kim S.Y., Ye B.R., Kim J., Ko S.C., Lee W.W., Kim K.N., Choi I.W., Jung W.K., Heo S.J. (2018). Anti-inflammatory effect of Apo-9’-fucoxanthinone via inhibition of MAPKs and NF-kB signaling pathway in LPS-stimulated RAW 264.7 macrophages and zebrafish model. Int. Immunopharmacol..

[48] Zhang L., Wang X., Si H. (2022). Synergistic anti-inflammatory effects and mechanisms of the combination of resveratrol and curcumin in human vascular endothelial cells and rodent aorta. J. Nutr. Biochem..

[49] Chen J., Li D.L., Xie L.N., Ma Y.R., Wu P.P., Li C., Liu W.F., Zhang K., Zhou R.P., Xu X.T. (2020). Synergistic anti-inflammatory effects of silibinin and thymol combination on LPS-induced RAW264.7 cells by inhibition of NF-κB and MAPK activation. Phytomedicine.

[50] Park G., Lee S.H., Han J.Y., Oh D.S. (2018). Altered TNF-α response by Aconibal^®^ and methotrexate in a lipopolysaccharide-induced setting of inflammatory conditions: Potential on a synergistic combination. J. Ethnopharmacol..

